# Effects of Oxytocin on Social Comparisons in Intergroup Situations

**DOI:** 10.3390/brainsci11091227

**Published:** 2021-09-17

**Authors:** Eun Young Kim, Sunhae Sul, Min Woo Lee, Kyung-Ok Lim, Na Young Shin, Sung Nyun Kim, Jun Soo Kwon, Hackjin Kim

**Affiliations:** 1 Department of Occupational Therapy, Soonchunhyang University, Asan-si 31538, Korea; eykim@sch.ac.kr; 2 Department of Psychology, Pusan National University, Busan 46241, Korea; ssul@pusan.ac.kr; 3 Department of Anthropology, Emory University, Atlanta, GA 30322, USA; minwoo.lee@emory.edu; 4 Department of Psychiatry, National Institute of Forensic Psychiatry, Ministry of Justice, Gonju-si 32621, Korea; kyungoklim77@gmail.com; 5 Department of Forensic Psychology, Kyonggi University, Suwon-si 16227, Korea; shinny@kyonggi.ac.kr; 6 Department of Psychiatry, Seoul Medical Center, Seoul 02053, Korea; zoouncle@gmail.com; 7 Department of Psychiatry, Seoul National University College of Medicine, Seoul 03080, Korea; kwonjs@snu.ac.kr; 8 Department of Psychology, Korea University, Seoul 02841, Korea

**Keywords:** oxytocin, social comparison, intergroup situation, in-group bias

## Abstract

Oxytocin (OXT) is known to affect various social processes, including social comparisons and intergroup competition. In this study, we examined whether social comparisons in intergroup situations can be modulated by OXT and, if so, how this modulation manifests. Using a double-blind placebo-controlled design, we randomly assigned male participants to either OXT or placebo treatment and then asked them to play a card game with either an in-group or an out-group member. The OXT-treated participants showed a greater social comparison effect in the games with an out-group member than in games with an in-group member. Specifically, the participants in the OXT treatment condition showed a greater acceptance rate for relative gain (downward comparison) and a lower acceptance rate for relative loss (upward comparison) while playing with an out-group member rather than an in-group member. In contrast, no such effect was observed among placebo-treated participants. These findings demonstrate that OXT facilitates intergroup social comparisons with out-group versus in-group members.

## 1. Introduction

When evaluating our own abilities or resources, we often compare ourselves with others [1]. We experience the same resource differently depending on who we compare ourselves to. For example, downward comparison with an inferior other leads to positive affect, while upward comparison with a superior other leads to negative affect [2]. Such a social comparison motivates people to reduce the difference between themselves and others in an upward comparison situation where they have fewer resources than others. For instance, people are less satisfied with a smaller salary than those of their colleagues or workers engaged in the same occupation in other companies [3] and tend to choose a job offering a lower salary but an equivalent level to that of others than a job providing a higher salary that is less than the salary of others [4]. People also tend to exaggerate the difference in a downward comparison situation where they have more resources than others. For instance, even young children at the age of six choose an absolutely smaller amount of resource that is relatively greater than others over another absolutely larger amount of resource that is equal between themselves and others [5]. These findings indicate that social comparison, which makes people focus more on their relative rather than absolute states, appears to be a fundamental part of the human mind [6,7] and can be an important determinant of the subjective utility that people experience from their choices.

Social comparison occurs not only between individuals but also between groups, such that an upward (or downward) comparison occurs when the in-group is inferior (or superior) to the out-group [8]. For instance, basketball fans report positive emotions toward the misfortune (e.g., injury) of their rivals and negative emotions toward the fortune (e.g., quick recovery) of their rivals [9]. In a competitive intergroup context, people often compare their in-group against the other (competing) group because the superiority of the out-group could mean the inferiority of the in-group [10]. Furthermore, the success of the out-group (i.e., upward comparison) increases activities in the anterior cingulate cortex, an area of emotional pain, whereas the failure of the out-group (i.e., downward comparison) elevates activities in the ventral striatum, an area of reward processing [11]. These studies provide behavioral and neural evidence that social comparisons may extend from the individual to the intergroup level.

Such an intergroup social comparison appears to be in line with the well-known phenomenon of in-group favoritism. Humans have evolved to form groups because a person in a group has a higher chance of survival than an individual alone, and a group has the advantage of achieving more difficult things such as hunting a big animal and defending its members from attacks through member cooperation [12]. In an intergroup situation, where one group competes against the other over limited resources, the success of the latter could be a threat to the former [13]. Humans may have evolved to become more prosocial toward in-group members and more defensive against out-group members to promote survival and fitness [14], although such propensity appears to be dependent on certain factors, such as social distance, personality traits, and situational context [15]. Accordingly, imagining common psychological/biological mechanisms underlying in-group favoritism and intergroup social comparisons may not be difficult.

Oxytocin (OXT), a neuropeptide produced in the parvocellular and magnocellular neurons of the hypothalamus and widely released into brain areas, including the limbic system and reward circuits [16,17,18], modulates social comparisons. For instance, OXT administration strengthens the emotional experience of envy and schadenfreude (i.e., pleasurable emotions toward the misfortune of another) in situations involving social comparisons [19]. Importantly, OXT modulates in-group bias [20]. For instance, OXT increases positive implicit attitudes toward in-group versus out-group by forming a strong association between the in-group and positive valence and a relatively weak link between the out-group and negative valence in the Implicit Association Test (IAT) [21]. Moreover, empathy toward in-group members enables people to cooperate within the in-group [20], and empathetic neural responses to pain delivered to in-group versus out-group members are differentially modulated by oxytocin receptor gene polymorphism [22] and intranasal OXT administration [23].

OXT also influences social decisions in situations of intergroup competition. For instance, in a competitive situation, such as the intergroup prisoner’s dilemma-maximizing difference (IPD-MD) game, OXT facilitates cooperation with in-group members while promoting non-cooperation or defensive aggression toward out-group members [24]. Given the well-known role of OXT in boosting interpersonal trust [25], OXT may facilitate an individual’s expectation for in-group members to cooperate in the IPD-MD game [24]. In-group favoritism could manifest itself as seeking strong in-group members that threaten a rival group, which increases a person’s chance of survival [26], and OXT increases the tendency to select a team member with a higher threat feature [27]. According to the social salience hypothesis, OXT regulates the salience of stimuli in a social context and attention to social stimuli [28]. Consistent with this hypothesis, OXT has been proposed to enhance the social salience of in-group information and intergroup situations [29,30]. Additionally, the effect of OXT on social behavior toward the out-group could be modulated by individual characteristics (e.g., xenophobic attitude) [31] and salience information (e.g., competition) [24].

Considering that competitiveness is related to comparison concerns [32] and that OXT affects intergroup competition [24], we can also hypothesize that OXT can influence intergroup social comparisons. However, whether such a modulatory role of OXT in interpersonal social comparisons can be extended to a situation of intergroup interaction remains unknown.

This study aimed to examine whether and how OXT differentially influences social comparisons in an intergroup situation using a double-blind, placebo-controlled design. Participants performed a social comparison task, playing a gamble-like card selection game with either an in-group or an out-group member, as used in our previous study [33]. We hypothesized that the intranasal administration of OXT would facilitate decision bias due to social comparisons when playing with an out-group versus an in-group member. We predicted that decisions based on social comparison would appear when the game partner was an out-group member rather than an in-group member and that such a difference would be more pronounced under OXT than placebo. In addition, we investigated whether a modulatory effect of OXT on social comparisons would be observed in downward or upward comparison situations or both.

## 2. Materials and Methods

### 2.1. Participants

Forty-two participants were recruited, but five were excluded due to alcohol or nicotine consumption within 12 h prior to the experiment (*n* = 3), misunderstanding of the procedure (*n* = 1), or OXT administration failure (*n* = 1). The remaining 37 male participants (19 OXT-treated participants, *M* = 23.94 years, *SD* = 2.42; 18 placebo-treated participants, *M =* 24.94 years, *SD* = 3.10) were randomly assigned to the OXT or placebo treatment group using a double-blind experimental design. We performed a power analysis using G*Power software to ensure that the final sample size was sufficient to obtain scientifically meaningful results [34]. Assuming a medium effect size of 0.06 and an alpha level of 0.05, to achieve a power of 0.90 for a mixed ANOVA with two groups and four repeated measurements, the G*Power analysis yielded a required sample size of 30, that is, 15 participants per group. All participants were healthy, took no medication, and had no mental disorders. This study was approved by the institutional review board of Korea University, and informed consent was obtained from all participants. All methods were performed in accordance with the relevant guidelines and regulations. The participants were compensated with KRW 18,000–24,000 (approximately 18–24 USD). The participants received KRW 15,000 as a base payment and additional or subtracted payment depending on the outcomes of the experiment.

### 2.2. Procedure

A cohort of three participants visited the laboratory at the same time in a single session. Only one of the three participants was included in the present experiment without knowing that the other two had been allocated to distinct studies. After providing informed consent, the participants were told that the experiments aimed to investigate the effects of OXT on decision making.

#### 2.2.1. Treatment

**Figure 1:**
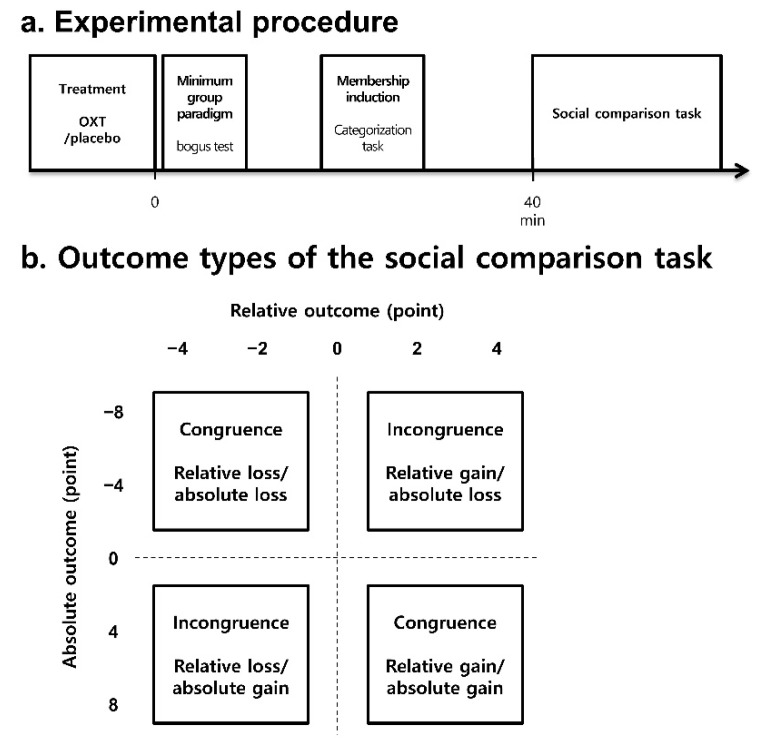
(**a**) Experimental procedure. Following the self-administration of OXT or placebo, the participants learned about in-group and out-group members via the minimal group paradigm and performed the categorization task designed for membership induction. Approximately forty minutes after the administration, they performed the social comparison task. (**b**) Outcome types of the social comparison task. The absolute outcomes ranged from −8 to +8, and the relative outcomes determined by the difference between the outcomes of the participants and those of the partners ranged from −4 to 4. The congruence condition included relative loss/absolute loss and relative gain/absolute gain trials, whereas the incongruence condition contained relative loss/absolute gain and relative gain/absolute loss trials.

Depending on the assigned condition, the participants were administered either a 40 IU/mL OXT solution or a sterile saline solution with an identical pH to that of the OXT solution [35]. The solution was prepared by the Seoul National University Hospital Pharmacy according to the protocol described by Marsh et al. [36]. We used a higher dose (40 IU) of the OXT treatment compared with the dose (24 IU) used in previous studies, as it has been reported to be effective in healthy Korean males [35]. The participants self-administered 10 puffs of OXT or placebo with a nasal spray providing 4 IU per pump while alternating nostrils. After each pump, the participants breathed deeply for 30–60 s. Approximately 40 min after the administration [37], the participants performed the social comparison task. During the waiting period, we manipulated group membership using the minimal group paradigm (Figure 1a) [38].

#### 2.2.2. Manipulation for Group Membership

The participants were given a paper-based bogus test asking them to answer questions about ambiguous figures, and the participants were then told that they would be assigned to the red or blue team based on their perceptual style, namely, a figure-based perception or a ground-based perception. However, the participant and another player were always assigned to a red team, and the other player was assigned to a blue team. The participants included in the present experiment were members of the red team regardless of their answers to the questions about ambiguous figures so that they could have both in-group (red team) and out-group (blue team) members as the targets of social comparisons. The participants were also told that all the participants in this study were assigned to one of two large groups, such that they were led to believe that each player was a member of a large group. Following the initial group assignment, the participants performed a short social categorization task known to facilitate group membership identification [38,39]. When the participants viewed a series of red or blue geometric shapes, ‘red team’ or ‘blue team,’ and the name of the in-group or out-group members, they had to categorize the stimuli by pressing a key for the in-group or another key for the out-group, as quickly and accurately as possible to earn incentives (KRW 1000) given to the winning team. The participants performed the social comparison task after completing the drug administration, the minimum group paradigm, and the membership induction procedure (Figure 1a).

#### 2.2.3. Social Comparison Task

**Figure 2:**
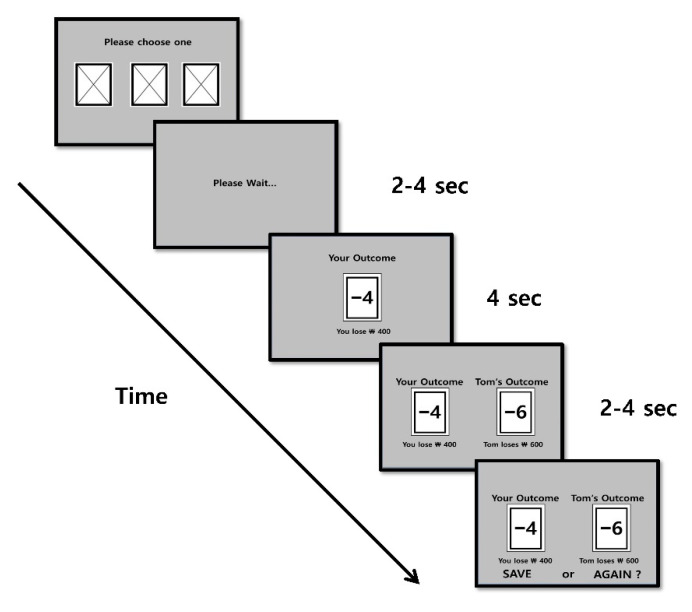
One representative trial of the social comparison task. In each trial, a participant is instructed to choose one of three cards. Two to four seconds after the choice, the outcome of the participant was presented for 4 s and followed by a display of the outcomes for the participant and the partner for 2–4 s. Thereafter, the participant was prompted to decide whether to save (accept) or repeat (reject) the current outcome.

The main task was the card selection game (Figure 2) adopted from the paradigm used by Kang et al. [33,40]. The participants were told that they would play the card game either with a member of their own team (the red team, i.e., in-group condition) or with the other team (the blue team, i.e., out-group condition) for 50 trials. At the beginning of each trial, the backs of the three cards were presented with an instruction to choose one. Two to four seconds after the participants chose a card, the score of the card and the amount of money the participants gained or lost (i.e., self-outcome) appeared for 4 s. Thereafter, the participants were presented with the score and monetary gain/loss of the card chosen by their playing partner (i.e., other outcomes) and the self-outcome to induce social comparisons among the participants. Two to four seconds later, the participants were asked whether they would accept their outcome or repeat the same trial once more after completing all the planned 50 trials. Repeated trials were excluded from the data analysis. They were told that the outcomes of five (10%) randomly selected trials would be added to or subtracted from the initial game money of KRW 5000. The entire task took approximately 15 min.

The participants were told that their decisions would only influence their outcomes and not the outcomes of their partners, and the same applied to the partner, although they could both watch the outcomes of every trial. This allowed us to measure decisions based on social comparisons separately from decisions related to social competition. The participants played the card game with an in-group member for 25 trials and an out-group member for another 25 trials. They were led to believe that the other two players performed the same task in separate rooms and that the outcome would be from −12 to 12. However, participants played alone and watched the predetermined outcomes that were designed to include five levels of absolute outcomes and five levels of relative outcomes (Figure 1b). Specifically, the outcomes of the participants ranged from −8 to +8 points with a four-point increment (five absolute levels): the points of absolute loss trials were −8 and −4, and the points of absolute gain trials were +8 and +4. The outcomes of the partners ranged from −12 to +12 points so that the differences between the outcomes of the participants and those of the partners could be −4, −2, 0, +2, and +4 (five relative levels). The difference points of the relative loss trials were −4 and −2, whereas those of the relative gain trials were +4 and +2. For instance, an outcome of −4 vs. −8 (self vs. other) was an absolute loss (earned point: −4) but a relative gain (difference point: +4), whereas an outcome of +4 vs. +8 was an absolute gain (earned point: +4) but a relative loss (difference point: −4). If social comparison is a major determinant of choice utility, then a participant would accept the relative gain/absolute loss and reject the relative loss/absolute gain. Assuming that genuine social comparisons would appear when the direction of the relative income was inconsistent with that of absolute income, the trials were divided into congruence (relative loss/absolute loss, relative gain/absolute gain) and incongruence (relative loss/absolute gain, relative gain/absolute loss) conditions. In the congruence condition, the decision based on the relative score was consistent with the decision based on the absolute score. For instance, if the outcomes for a participant and a partner were +4 and +2, respectively, then the participant would accept the outcome because it is an absolute and relative gain. Likewise, if the outcomes for a participant and a partner were −4 and −2, respectively, then the participant would reject the outcome because it is an absolute and relative loss. However, in the incongruence condition, a decision based on the relative score incurs a cost equal to the absolute score. For instance, if the outcomes for a participant and a partner were +4 and +8, respectively, then rejection based on relative loss incurs a cost of an absolute gain of 4. Likewise, if the outcomes for a participant and a partner were −4 and −8, respectively, then acceptance based on relative gain incurs a cost of an absolute loss of 4. Therefore, decisions due to social comparisons in the incongruence condition are bound to incur a cost equal to the value of the absolute outcomes.

#### 2.2.4. Protocol after Social Comparison Task

Upon completing the social comparison task, the participants evaluated the in-group and out-group members in terms of attractiveness, proximity, and similarity using a 7-point Likert scale. The impression scores were calculated by summing attractiveness, proximity, and similarity and were entered into a two-way mixed ANOVA with treatment (OXT and placebo) as a between-subjects factor and group membership (in-group and out-group) as the within-subject factor. No significant main effect of treatment or group membership, and no significant interaction effect of treatment by group membership on impression was observed, *Fs* < 0.27, *ps* > 0.612 (OXT in-group, *M* = 10.42, *SD* = 3.19; OXT out-group, *M* = 10.53, *SD* = 3.13; placebo in-group, *M* = 11.17, *SD* = 3.71, placebo out-group, *M* = 10.67, *SD* = 3.80).

During the debriefing, 15 participants reported that they did not know their assigned condition, and the rest of them guessed the condition at the chance level. Among the 37 participants, six reported suspicions about the cover story. We analyzed our data with or without these six participants and confirmed that the main finding of the role of OXT in intergroup social comparisons remained unchanged. Therefore, we included these participants in all subsequent analyses.

We measured individual characteristics related to variables of interest (i.e., social comparison and in-group favoritism) in order to test the difference between OXT- and placebo-treated participants and analyze the main analyses with these individual measures as covariates. After the experiment, the participants were asked to complete questionnaires measuring individual characteristics related to social comparison [41,42], such as the dispositional envy scale [42] and self-esteem scale [43]. Participants also responded to measurements related to in-group favoritism [44,45,46], such as the social dominance orientation scale [47], the individualism and collectivism scale [48], and the interpersonal reactivity scale [49]. Individual characteristics did not significantly differ between the OXT and placebo groups, *ts* < 1.38, *ps* > 0.178, except that the placebo-treated participants had higher collectivism than the OXT-treated participants, *t*(35) = 2.60, *p* = 0.013.

### 2.3. Data Analysis

In the main social comparison task, the levels of relative outcome were orthogonalized against the levels of absolute outcome to increase the chances of detecting social comparative behavior while avoiding a possible ceiling and floor effect. Thus, for our main analysis, we divided the trials into congruence and incongruence trials with respect to the discrepancies between the absolute and relative outcomes (Figure 1b).

To quantify the degree of social comparisons for each participant, we calculated the SCI by averaging across the acceptance rate in the relative gain trials and the rejection rate (1—the acceptance rate) in the relative loss trials, separately for each condition.

(1) $$\mathrm{SCI}=\frac{\mathrm{acceptace}\;\mathrm{rate}\;\mathrm{of}\;\mathrm{the}\;\mathrm{relative}\;\mathrm{gain}\;+\left(1-\mathrm{acceptance}\;\mathrm{rate}\;\mathrm{of}\;\mathrm{the}\;\mathrm{relative}\;\mathrm{loss}\right)}{2}$$

Thus, the SCI indicates the extent to which each participant accepted the relative gain and rejected the relative loss. In the congruence condition, there is no cost of accepting the relative gain and rejecting the relative loss; thus, the SCI should be close to one. In contrast, in the incongruence condition, where the relative gain means absolute loss and the relative loss means absolute gain, a greater SCI score indicates a greater tendency for social comparisons. This means that the participants should accept the relative gain even with absolute loss and/or reject the relative loss, even with absolute gain. Individual SCIs were entered into a three-way mixed ANOVA with treatment (OXT and placebo) as a between-subjects factor and group membership (in-group and out-group) and congruency (congruence and incongruence) as the within-subject factors. We performed ANCOVA with the individual characteristics of the participants (i.e., self-esteem, dispositional envy, social dominance orientation, collectivism, perspective taking, and empathetic concern) and the impression ratings of in-group and out-group members as covariates to confirm the result of ANOVA by controlling for the potential interaction effects of individual differences and the impressions of other players. Thereafter, we further analyzed incongruence trials in which we expected social comparisons to be most pronounced. We performed non-parametric Wilcoxon signed-rank tests to confirm the results. The significance level alpha was set at 0.05.

## 3. Results

We used a three-way mixed ANOVA with factors of treatment (OXT and placebo), group membership (in-group and out-group), and congruency (incongruence and congruence) to test the individual SCIs, to examine whether OXT differentially influenced social comparisons with in-group versus out-group members in the incongruence versus congruence condition trials.

**Figure 3:**
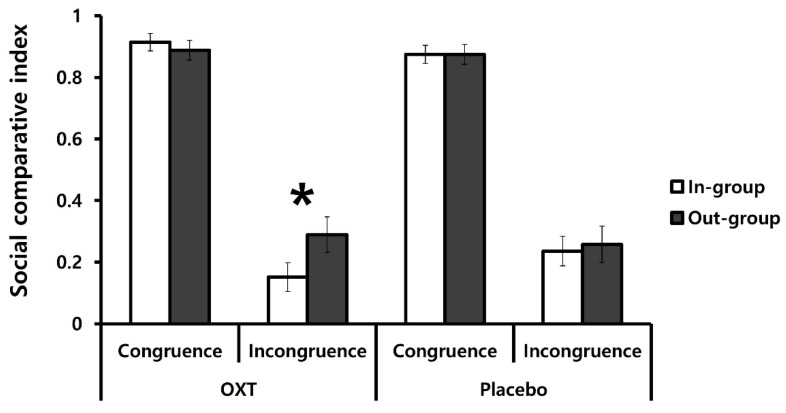
The mean social comparative indices across conditions. Error bars represent the standard errors of the mean for each condition. OXT enhanced differences in the social comparative index between the in-group and out-group conditions only in the incongruence condition (* *p* < 0.05).

As predicted, this analysis revealed a significant three-way interaction among treatment, group membership, and congruency, *F*(1, 35) = 5.30, *p* = 0.027, *η*2 = 0.13 (Figure 3, Table 1). This three-way interaction effect was confirmed by an ANCOVA with the individual characteristics of the participants and the impression ratings to the in-group/out-group members included as covariates, *F*(1, 27) = 4.77, *p* = 0.038, *η*2 = 0.15. Under OXT, the interaction between congruency and group membership was significant, *F*(1, 18) = 8.45, *p* = 0.009, *η*2 = 0.32. Planned comparisons revealed that, under OXT, the SCI in the incongruence condition was significantly higher in the out-group than in the in-group conditions, *t*(18) = 2.41, *p* = 0.026, *d* = 0.55, whereas no significant difference was observed in the SCI for the congruence condition between the out-group and in-group conditions, *t*(18) = 0.89, *p* = 0.385. Non-parametric Wilcoxon signed-rank tests confirmed the results of the incongruence (*Z* = 2.39, *p* = 0.017) and congruence (*Z* = 0.81, *p* = 0.417) conditions. In contrast, the interaction between congruency and group membership was not significant for placebo treatment, *F*(1, 17) = 0.81, *p* = 0.381. In sum, the significant three-way interaction effect was primarily driven by higher social comparative behavior toward the out-group from an in-group partner in the incongruence condition under the OXT treatment.

The main effect of congruency was also significant, *F*(1, 35) = 245.35, *p* < 0.001, *η*2 = 0.88. The SCI was higher in the congruence (*M* = 0.89, *SD* = 0.12) than in the incongruence (*M* = 0.23, *SD* = 0.21) conditions. While this result may seem counterintuitive, it reflects the tendency of the participants to follow absolute outcomes, which were congruent with relative outcomes in the congruence condition. No other main effects were significant for treatment, *F*(1, 35) = 0.00, *p* = 0.998; for group membership, *F*(1, 35) = 2.71, *p* = 0.108. The two-way interaction between congruency and group membership was significant, *F*(1, 35) = 8.82, *p* = 0.005, *η*2 = 0.20. Planned comparisons further revealed that the effect of group membership on social comparison was significant in the incongruence condition (out-group condition: *M* = 0.27, *SD* = 0.25; in-group condition: *M* = 0.19, *SD* = 0.21; *t*(36) = 2.49, *p* = 0.018, *d* = 0.41), whereas in the congruence condition, no significant effect of group membership emerged (out-group condition: *M* = 0.88, *SD* = 0.14; in-group condition: *M* = 0.90, *SD* = 0.12; *t*(36) = 0.78, *p* = 0.440). Non-parametric Wilcoxon signed-rank tests also validated the results of incongruence (*Z* = 2.59, *p* = 0.01) and congruence (*Z* = 0.68, *p* = 0.499) conditions. No other two-way interactions were significant for interaction between treatment and congruency, *F*(1, 35) = 0.39, *p* = 0.534; for interaction between treatment and group membership, *F*(1, 35) = 1.28, *p* = 0.266.

We also performed an exploratory analysis to determine whether OXT had differential effects on upward and downward intergroup social comparisons in the incongruence condition. As the intergroup difference was our main interest, we calculated an intergroup difference score for each individual by subtracting the acceptance rate of the in-group condition from that of the out-group condition. A 2-by-2 mixed ANCOVA with the treatment (OXT and placebo) as a between-subjects factor, direction (downward and upward) as a within-subject factor, and the individual characteristics of the participants and the impression ratings to the in-group/out-group members included as covariates on the difference scores revealed a marginally significant interaction effect, *F*(1, 27) = 4.19, *p* = 0.050, *η*2 = 0.13. Planned comparisons also revealed that, under OXT, the difference score was significantly higher in the downward (*M* = 0.15, *SD* = 0.25) than in the upward (*M* = −0.13, *SD* = 0.29) directions, *t*(18) = 2.41, *p* = 0.027, *d* = 1.06, whereas under placebo, no significant difference existed between the downward (*M* = 0.01, *SD* = 0.14) and upward (*M* = −0.03, *SD* = 0.08) directions, *t*(17) = 0.90, *p* = 0.381. Non-parametric Wilcoxon signed rank tests further confirmed the results of the OXT (*Z* = 2.39, *p* = 0.017) and placebo (*Z* = 0.97, *p* = 0.334) conditions.

**Figure 4:**
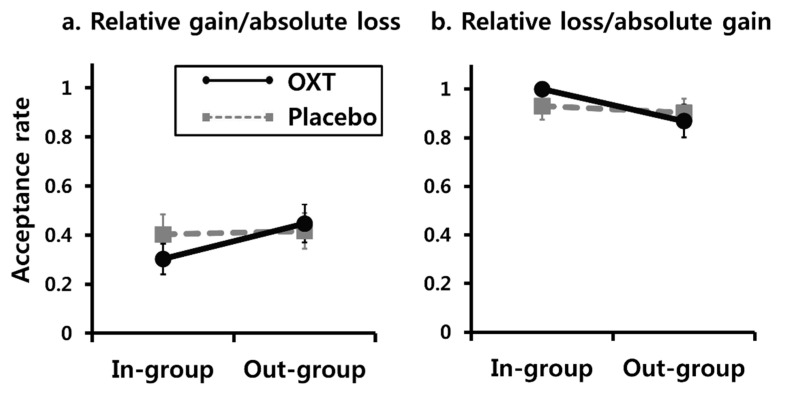
The mean acceptance rates across conditions in the incongruence condition separately displayed for (**a**) the relative gain/absolute loss trials (i.e., downward comparison) and (**b**) the relative loss/absolute gain trials (i.e., upward comparison). Error bars represent the standard errors of the mean for each condition.

We further analyzed the difference in acceptance rates between the in-group and out-group for each direction of social comparisons in the incongruence condition (Figure 4). For a downward comparison, the participants treated with OXT were more likely to accept the outcomes when the other player was an out-group member (*M* = 0.45, SD = 0.34) rather than an in-group member (*M* = 0.30, *SD* = 0.27), *t*(18) = 2.48, *p* = 0.023, *d* = 0.57, whereas those treated with placebo did not show differences between the out-group (*M* = 0.42, *SD* = 0.31) and in-group (*M* = 0.40, *SD* = 0.34) conditions, *t*(17) = 0.44, *p* = 0.668. Non-parametric Wilcoxon signed-rank tests also verified the results of the OXT (*Z* = 2.21, *p* = 0.027) and placebo (*Z* = 0.45, *p* = 0.655) conditions. For upward comparison, there was a trend whereby OXT-treated participants tended to discard the outcomes more when the other player was in the out-group (*M* = 0.87, *SD* = 0.29) than in the in-group (*M* = 1, *SD* = 0.00), *t*(18) = 1.96, *p* = 0.066, *d* = 0.45, whereas placebo-treated participants did not show differential acceptance rates with respect to the other players’ group membership (in-group condition: *M* = 0.93, *SD* = 0.24; out-group condition: *M* = 0.90, *SD* = 0.25), *t*(17) = 1.46, *p* = 0.163. Non-parametric Wilcoxon signed-rank tests validated the results of the OXT (*Z* = 1.83, *p* = 0.068) and placebo (*Z* = 1.41, *p* = 0.157) conditions.

In sum, the participants treated with OXT showed higher social comparisons with the out-group versus in-group members in the incongruence condition, whereas no such difference due to group membership was found in the congruence trials. Conversely, the participants treated with placebo did not react differently according to the group membership of the other player in any situation. These results indicate that OXT treatment facilitated social comparisons in the intergroup situation, particularly when relative and absolute incomes differed.

## 4. Discussion

The current study aimed to examine whether OXT modulates social comparisons in the minimal intergroup situation. The findings demonstrated that OXT facilitates social comparisons with out-group versus in-group members. Specifically, the participants treated with OXT were more likely to accept relatively fewer losses and tended to reject relatively fewer gains in the game with an out-group member than an in-group member. In contrast, those treated with a placebo did not show altered behavior between the out-group and in-group conditions. Such an OXT-induced increase in social comparative decisions against out-group versus in-group members is consistent with previous findings on the role of OXT in in-group favoritism [20] and parochial altruism [23]. The present study is particularly meaningful in that it provides a link between the previously reported role of OXT in in-group bias [19,50,51,52] and the role of OXT in interpersonal social comparisons [18], which may provide crucial insights into the fundamental psychological motive underlying in-group bias.

Social comparisons with the out-group versus the in-group were observed only in incongruent situations (i.e., the relative loss/absolute gain and the relative gain/absolute loss trials). Importantly, this in-group bias during incongruent situations was observed only in the OXT treatment. The OXT-treated participants were more likely to accept relatively smaller losses and to reject relatively smaller gains when playing with an out-group member. This OXT effect was significant in the downward comparison situation and only marginally significant in the upward comparison situation. Consistent with these findings, OXT has been shown to increase envy and schadenfreude in interpersonal situations [18], although the downward comparison for the out-group may be more pronounced by OXT in an intergroup situation.

Although no statistically significant results were observed in the upward comparison situation, it is noteworthy that the OXT-treated participants in this situation accepted all the outcomes when playing with an in-group member. This demonstrates that OXT-treated participants were more likely to avoid inferiority to an out-group member but not an in-group member. A future study may be essential to confirm this statistically weak but highly interesting behavioral finding.

Although a majority of previous studies have reported a primary role of OXT favoring in-group bias, an opposite role of OXT has also been reported in more recent reports [53,54]. For instance, in the intergroup cooperation context, OXT facilitates cooperation toward an out-group as well, and compared with the placebo-treated participants, the OXT-treated participants contributed more resources to the out-group and the in-group [53]. OXT also increases empathy toward out-group members. OXT-treated Jewish participants showed similar empathy for Palestinian-Arab suffering and Jewish suffering, whereas placebo-treated Jewish participants showed a larger empathetic response to the pain of an in-group member [54]. Moreover, the participants who showed increased endogenous OXT levels offered an identical split proposal to the in-group and out-group in an ultimatum game [55]. These studies seem to contradict the findings of the current study, which shows the effect of OXT in differentiating between the in-group and out-group. However, such inconsistencies between studies could be resolved by the social salience hypothesis of the OXT function [27,28,29]. This hypothesis suggests that OXT plays a key role in enhancing attitudes and behaviors according to social cues with higher importance/salience in a given situation [27]. Based on this hypothesis, OXT can promote altruism toward or defense against an out-group, depending on which information is highlighted and becomes salient. For instance, when universality is emphasized or when harm aversion is made salient, OXT can facilitate cooperation and empathy regardless of group membership. In contrast, when competition between groups is made salient, OXT would increase in-group favoritism. By repeatedly reminding the participants of their group identity and simultaneously presenting their own outcomes with those of other members, the experimental setup of the present study may have naturally induced motivation for group competition, which was made more salient with the OXT treatment.

The present study can be distinguished from previous studies on social comparisons in two respects. First, unlike in previous studies where participants were asked to explicitly report their social comparison emotions (i.e., envy and schadenfreude) using the Likert scale, in the present study, the participants were simply asked to decide whether to accept or reject the outcomes without any explicit instructions alluding to social comparisons. Such a design is necessary to minimize potential demand characteristics. Second, in the present study, the participants’ decisions during the card game did not affect the outcomes of the other players. We believe that such a design is helpful in the measurement of pure social comparative decisions without contamination due to some other motivations, such as group competition and utility maximization.

Recent studies have demonstrated that OXT increases in-group favoritism but not necessarily out-group hatred in a situation of intergroup competition [19,20,23]. Consistent with these findings, the OXT-induced difference in social comparisons with in-group versus out-group members may have been caused by reduced social comparisons with in-group members rather than increased social comparisons with out-group members. Alternatively, OXT may have facilitated social comparisons with out-group members by decreasing interpersonal trust toward out-group members. Supporting this view, the amygdala shows greater activation in response to the faces of out-group versus in-group members [56], and acute stress, to which the amygdala is particularly responsive [57], reduces trustworthiness toward out-group members rather than in-group members [58]. Considering the recent report that the amygdala response to fearful stimuli increased rather than decreased with high doses of OXT (i.e., 48 IU) [59], we can infer that the dose (i.e., 40 IU OXT) used in the current study may have increased amygdala activity, leading to enhanced social comparisons with out-group versus in-group members through selective decreases in trust toward out-group versus in-group members. In fact, when we compared the SCI of the incongruence condition between the OXT- and placebo-treated participants, we found no significant OXT-induced change in social comparisons either in the in-group condition, *t*(35) = 1.27, *p* = 0.214, or in the out-group condition, *t*(35) = 0.39, *p* = 0.698. However, these results do not provide an unequivocal answer to whether OXT affects social comparisons with the in-group, out-group, or both, partly because of the between-subjects design used in the present study. Future studies using a within-subject design, in which the same participant receives the OXT and placebo treatments, would be useful in determining the direction of the OXT effect in different intergroup contexts.

The results of the present study do not imply a different level of social comparison between the in-group and the out-group among the participants treated with placebo. This finding appears to be inconsistent with a previous report showing that people, even without OXT, feel heightened pleasure when watching the failure of the out-group in the context of intergroup competition (e.g., a baseball game) [10]. This difference between our study and the previous study has at least two possible explanations. First, in the present study, decisions to accept or reject the outcomes did not affect the outcomes of counterpart players. Second, we relied on laboratory-induced group membership (i.e., minimal group paradigm) rather than pre-existing group membership in real life. Due to these differences, the participants in the present study may have been only weakly motivated to engage in intergroup competition unless they were treated with OXT. Therefore, examining whether and how intergroup social comparisons can be modulated by certain factors, such as the strength of group membership and the degree to which it can contribute to the goal of the in-group versus the out-group, is of great interest.

Only men were included in the current study to avoid any complexity due to fluctuations in plasma oxytocin and sex hormones across the menstrual cycle of women [60]. Indeed, several studies have reported potential sex differences in OXT administration. For instance, women showed less sensitivity to intranasal OXT administration than men, probably because women already have high levels of OXT affected by estrogen [61,62], and OXT administration tended to increase in-group favoritism in men more than in women [52]. Taken together, we speculate that the OXT-induced change in social comparisons in intergroup situations would be weaker in women.

This study has several limitations that lead to further research. First, we did not measure changes in mood and state anxiety, which are typically measured in OXT administration studies. However, we believe that this limitation is not crucial in the interpretation of the findings of this study because all the factors, except for the OXT treatment, were within-subject factors. Therefore, all the results from this study cannot be simply attributed to a general mood change or anxiety reduction following OXT treatment, considering that the same participants treated with OXT reacted differently to the group membership of the other players. Future research may be crucial to address exactly how mood changes interact with oxytocin effects. Second, there was a possibility that a greater number of competitive participants were assigned to OXT treatment by chance, and they were more primed for competition during the categorization task. Although we did not include a direct measure of competitiveness [63], we believe that we could rule out such a possibility because there was no difference between OXT-and placebo-treated participants in social dominance orientation, which is also known to reflect competitiveness in intergroup situations [64]. Third, given that oxytocin facilitates recognition of social stimuli [65], it is possible that oxytocin could improve group recognition in the categorization task, which could then be related to increased social comparison in the intergroup situation. Thus, the reaction time data from the categorization task might have provided useful indices of the performance of group identification. Unfortunately, however, we did not obtain response data from the categorization task because it was included for manipulation purposes only. A future study would be necessary to examine the effect of oxytocin on group identification. Fourth, the reaction time of the social comparison task was also not available because we planned to extend this study to an fMRI study, which will require temporal jittering between the presentation of social comparative information and a button press for statistical decomposition between the events due to delayed hemodynamic responses. Future studies are necessary to examine the effects of social comparative information on reaction time. Finally, this study included only healthy male participants but could be extended to include female participants as well as people with psychiatric disorders.

## 5. Conclusions

The present study demonstrates that intra-nasally administered OXT treatment can facilitate social comparisons with out-group versus in-group members even in a minimal intergroup situation, which would not normally be otherwise displayed. We believe that the present study provides important insights into our understanding of the psychological and biological mechanisms underlying intergroup behaviors stemming from in-group favoritism.

## Figures and Tables

**Table 1:** Mean (SD) social comparative indices for each condition.

	OXT		Placebo	
	Congruence	Incongruence	Congruence	Incongruence
In-group	0.91 (0.09)	0.15 (0.14)	0.88 (0.15)	0.24 (0.26)
Out-group	0.89 (0.13)	0.29 (0.26)	0.87 (0.15)	0.26 (0.24)

## Data Availability

The data that support the findings of this study are available from the corresponding author upon reasonable request.
